# Brief use of a specific gun in a violent game does not affect attitudes towards that gun

**DOI:** 10.1098/rsos.160310

**Published:** 2016-11-23

**Authors:** Joseph Hilgard, Christopher R. Engelhardt, Bruce D. Bartholow

**Affiliations:** 1 Annenberg Public Policy Center, University of Pennsylvania, PA, USA; 2 CARFAX, Inc., Columbia, MO, USA; 3 Psychological Sciences, University of Missouri, Columbia, MO, USA

**Keywords:** media violence, video games, product placement, firearms, public policy, Bayesian analysis

## Abstract

Although much attention has been paid to the question of whether violent video games increase aggressive behaviour, little attention has been paid to how such games might encourage antecedents of gun violence. In this study, we examined how product placement, the attractive in-game presentation of certain real-world firearm brands, might encourage gun ownership, a necessary antecedent of gun violence. We sought to study how the virtual portrayal of a real-world firearm (the Bushmaster AR-15) could influence players' attitudes towards the AR-15 specifically and gun ownership in general. College undergraduates (*N* = 176) played one of four modified video games in a 2 (gun: AR-15 or science-fiction control) × 2 (gun power: strong or weak) between-subjects design. Despite collecting many outcomes and examining many potential covariates and moderators, experimental assignment did little to influence outcomes of product evaluations or purchasing intentions with regard to the AR-15. Attitudes towards public policy and estimation of gun safety were also not influenced by experimental condition, although these might have been better tested by comparison against a no-violence control condition. By contrast, gender and political party had dramatic associations with all outcomes. We conclude that, if product placement shapes attitudes towards firearms, such effects will need to be studied with stronger manipulations or more sensitive measures.

## 1. Introduction

Although much attention has been paid to the question of whether violent video games increase aggressive behaviour, little attention has been paid to how such games might encourage antecedents of gun violence. One such potential antecedent is gun ownership. Possession of a firearm is, after all, necessary to commit gun violence. In their report to the National Science Foundation on what is known and what needs to be known about youth violence, Bushman *et al*. [1] comment that youths exposed to smoking or drinking media characters are more likely to start smoking or drinking themselves. They suggest that ‘research should test whether youth are more interested in acquiring and using guns after exposure to movie characters that use guns’ [1, p. 32]. In line with that suggestion, this study examined whether exposure to violent video games might foster interest in the acquisition of guns.

To date, little research has examined how first-person-shooter video games may influence attitudes towards firearms. One correlational study reports that college undergraduates who have been exposed to more violent video games have more negative attitudes towards gun control legislation [2]. This same report cites an unpublished study ([3], a non-experimental dissertation) on a modest sample (*N* = 78) of adolescents. This latter study found a significant correlation between violent video game exposure and reduced evaluations of the seriousness and deserved punishment for playing with guns. However, correlations between similar predictors and similar outcomes were not statistically significant; for example, violent television exposure was not significantly associated with any outcome, and the relationship between violent video game exposure and evaluations of guns fell just short of statistical significance. The relationship seems, therefore, somewhat inconsistent and in need of further study.

Despite the dearth of published research on this question, previous research and theory give us reason to expect that violent video games could influence attitudes toward firearms. The general learning model [4] posits that video games can act as a learning tool, and that schema and associations learned within a game may influence behaviours and attitudes outside the game. Thus, if a violent video game makes a particular gun look attractive or rewards players' use of a particular gun, players might be expected to have more positive attitudes towards that gun following gameplay.

### 1.1. Product placement

Product placement is an advertising technique that attempts to harness just that process. In product placement, a brand or product is integrated into the entertainment media experience. That is, in contrast with a self-contained advertisement presented during a commercial break, product placement attempts to change brand attitudes by making the brand part of the entertainment. Such advertising is suspected of being especially effective. Because product placement is not explicitly an advertisement, it may circumvent viewers' resistance to overt marketing [5]. Product placement may also leverage the audience's attitudes towards the characters to influence brand attitudes. For example, a favoured character may prefer a certain branded product. In this way, viewers' positive attitudes towards the character may create positive attitudes towards the brand [6].

Video games are hypothesized to be especially effective for product placement due to their interactive nature. Examples are plentiful. Sometimes in-game products are strictly incidental to gameplay. For example, Barack Obama's 2008 presidential campaign took out ads on billboards in the game *Burnout: Paradise*. Players would see the billboards alongside the highway as they drove, but would not have to interact directly with the advertisement. At other times, in-game products allow players to customize their avatar using real-world fashion. Expansion packs for The Sims allowed players to dress their characters in H&M clothing and furnish their homes with IKEA furniture. Skaters in *Tony Hawk* games can wear real-world fashion brands and ride branded skateboards. In other cases, the use of placed products is tied to gameplay-relevant rewards. In *Alan Wake*, players could earn an achievement by watching an advertisement for Verizon Wireless on a TV within the game. In *Oddworld: Munch's Oddysee,* players could recover health by drinking SoBe Lifewater, a branded soft drink. *SimCity 5* offered players the opportunity to place charging stations for the Nissan LEAF electric car. These charging stations made the virtual citizens happy, encouraging virtual mayors to place them. These latter examples could be expected to be more influential than the previous examples, as the branded product directly helps the player to achieve in-game goals.

A number of studies report evidence of the efficacy of in-game product placement, but paradigms and outcomes are heterogeneous. Results are also sometimes inconsistent across outcomes within a single study, so the robustness of the phenomenon is still uncertain. Glass [7] reports that players of the game *Fight Night 3* were quicker to pair in-game brands with the concept ‘good’ in an implicit association task than they were control brands not seen in the game. Mackay *et al*. [8] report that participants assigned to drive a Holden Monaro in the game *Gran Turismo* were more likely to remember the brand. Yang *et al.* [9] report that exposure to brands within a video game increased their accessibility in both implicit and explicit memory tasks.

Thus, there is some experimental evidence for the efficacy of video game product placement in shaping brand attitudes, awareness and recall, but the evidence is modest and piecemeal. Moreover, none of these inspect how video game product placement might influence attitudes towards firearms.

### 1.2. Firearms and video games

Although the research underlying product placement is modest, these advertising practices are the subject of both consumer and media scrutiny. One particular source of scrutiny is the use of real-world firearms in popular video games, a practice that some consider a potentially hazardous promotion of the ownership and use of deadly weapons.

Such product placement became the subject of international media attention when a military-themed first-person-shooter game, *Medal of Honor: Warfighter* (similar to the bestselling *Call of Duty* series), made explicit and strong marketing ties to weapons manufacturers and sales. *Warfighter*'s marketing made much of the purported authenticity of the game's content, featuring settings taken from real-world American military operations. As part of this marketing campaign, *Warfighter* hosted a website that listed all the guns the player could use in the game and linked to their real-world manufacturers. Players could order special editions of knives and a tomahawk licensed with the *Warfighter* brand, or even order a particular gun to be shipped to their local federally licensed firearm dealer [10]. *Warfighter* faced criticism over the explicit association between in-game violence and the promotion of real-world weapons, and later the special-edition tomahawk and online gun store were taken down [11,12].

This event led the media to more closely scrutinize the associations between the marketing of real-world and virtual firearms. Some media outlets argued that in-game representations of real-world guns were a powerful marketing force. Eurogamer argued that the portrayal of guns in games is a source of both revenue and free advertising for firearms manufacturers, as the manufacturer both collects a licensing fee and enjoys increased brand awareness from the product placement [13].

Eurogamer further argued that firearms manufacturers have substantial control over their products' in-game portrayals, quoting a Barrett Firearms representative as saying ‘We want to know explicitly how the rifle is to be used, ensuring that we are shown in a positive light […] such as the “good guys” using the rifle. [The gun must] perform to the standards that our rifles do in the real world. Barrett Firearms is known for its quality and the brand must always be placed on that foundation.’ ([13], para. 25) This quote implies that the in-game quality of the firearm is important: a powerful weapon may become more desirable, whereas an ineffective weapon may become less desirable.

As part of their coverage, Eurogamer interviewed Anthony Toutain, a representative for Cybergun, a company that manufactures BB guns and manages licensing arrangements for video games and firearms. Toutain reported that attractive in-game portrayals increase the demand for real-world replicas:

We definitely see sales of particular [BB] guns increase when they are featured in popular video games, such as Call of Duty. For example, sales of the FAMAS [French rifle] exploded in the US when Call of Duty decided to use it as one of the best weapons in the game.

Before then children in America [didn't] want to buy the FAMAS airsoft gun, simply because they don't know this brand. But when they play every day with a new brand in a video game, finally they want to buy it in reality. The sales increase can be enormously significant.

[13, paras. 42–43]

In the light of this phenomenon, we wanted to study the process by which in-game representations of firearms could influence perceptions of their real-world counterparts. If the gun seemed powerful and accurate in the game, would it seem that way outside the game?

We hypothesized that playing a video game featuring a powerful, attractive rendition of a real-world firearm would increase perceptions that the real-world counterpart is powerful, effective and desirable. These effects were hypothesized to hold relative to a control condition in which the game features a weak, unattractive rendition of the real-world firearm, as well as a condition in which the game does not feature the real-world firearm.

## 2. Material and methods

We report how we determined our sample size, all data exclusions (if any), all manipulations and all measures in the study. All data, measures, materials and procedures are available as a GitHub repository at https://github.com/Joe-Hilgard/VVG-product-placement.

### 2.1. Participants

Participants were 176 college undergraduates (122 male, 53 female) at a large Midwestern university participating for partial course credit. We had hoped to collect data from 50 people per cell, but the semester ended before the target sample size was reached. All provided written consent.

The study was advertised as a study of political attitudes. Each session lasted approximately 30 min. All research activity was reviewed and approved by the University of Missouri's institutional review board (Project number 1 207 000, Review ID 111 749). Participants were chiefly White (78%), with some African-American (13%), Asian (3%) and bi-racial (5%) participants. Three per cent identified as Latino.

### 2.2. Procedure

The study design was a 2 (Character's gun: AR-15 or plasma rifle) × 2 (gun power: strong or poor) between-subjects design. Subjects were assigned to one of the four conditions based on their subject number. (e.g. subjects 1, 2, 3, 4 and 5 were assigned to conditions 1, 2, 3, 4 and 1. Experiments were not conducted in multiples of 4 per day or 4 per week, so results are not confounded by time-of-day or day-of-week effects.) Cell sizes were balanced: 45 played the strong AR-15 game, 43 played the strong plasma rifle game, 44 played the weak AR-15 game, and 44 played the weak plasma rifle game. The procedure is described below. Research assistants worked from a script.

Participants were asked whether they would prefer the mouse *y*-axis to be inverted or not (i.e. whether they would pull the mouse towards themselves to look up or whether they would push the mouse away to look up). The research assistant adjusted this preference accordingly in the game's control settings.

Participants played the game for 15 min. As a manipulation check, the game was programmed to log the number of enemies the player killed and the number of times the player died. At the end of the game, the research assistant pushed a button to display these logged numbers and recorded them. Games were quit directly to desktop by pressing ALT-F4.

After playing the game, participants filled out a paper questionnaire. Upon completion of this questionnaire, participants were thanked and debriefed.

### 2.3. Measures and manipulations

#### 2.3.1. Video game

Participants played one of four modified versions of the first-person-shooter video game *Doom*. Players had to navigate a series of levels, fighting through zombies and demons. Enemies would try to bite the player or to shoot the player with guns or fireballs. The player had to shoot the enemies, pick up health and ammo power-ups, and make it to the end of each level. If the player took too many wounds, the player's health would be depleted and the level would have to be attempted again.

To ensure that participants spent as much time as possible using the gun, levels were custom-designed to be easy to navigate. This way, participants would spend as much time as possible in combat and as little time as possible exploring the level or being lost.

#### 2.3.2. Character's gun

In each version of the game, the player-character had only a single gun. In the *AR-15* condition, this was a realistic rendition of the AR-15 Bushmaster rifle, a popular home-defence and general-purpose rifle. Like its real-world counterpart, the virtual AR-15 was a semi-automatic rifle (e.g. it fired one round at a time, but did not need to be manually rechambered between rounds) with a 20-round magazine. When the player fired the rifle, it would fire at a steady pace; after 20 rounds, it would have to be reloaded.

In the *plasma rifle* condition, the player-character instead had a science-fiction rifle we called the ‘Martian ZQ-5 Plasma Rifle.’ Its properties (e.g. rate of fire, damage per bullet, rounds per magazine, accuracy) were identical to the virtual AR-15.

**Figure 1.**
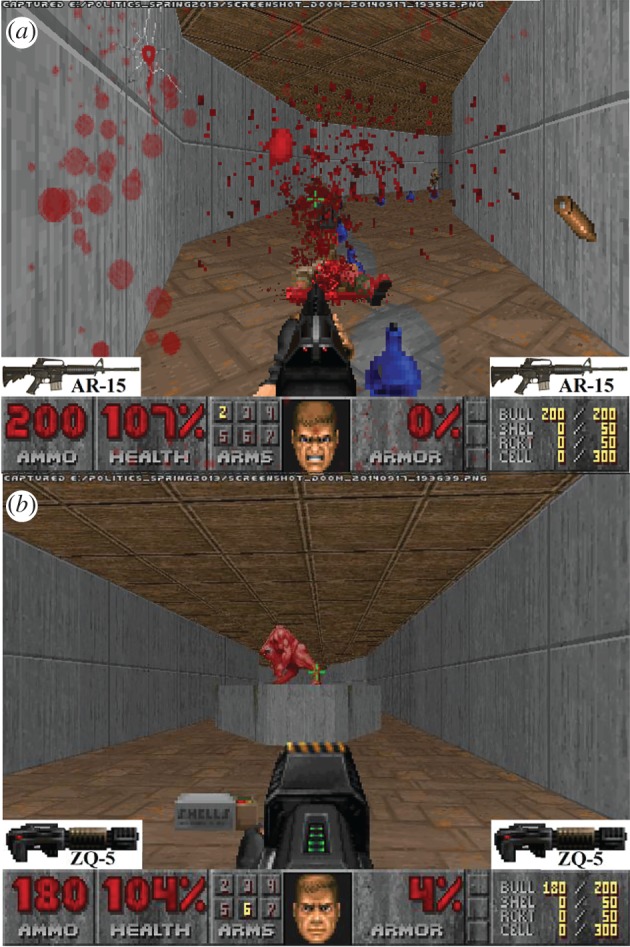
Screenshots from the AR-15 condition (*a*) and ZQ-5 condition (*b*).

To strengthen the manipulation, a description of the assigned gun was given in the cover story. Moreover, a picture-in-picture icon of the gun and its name was presented bilaterally on the game screen (figure 1).

#### 2.3.3. Gun power

To make the player's gun more or less desirable, its in-game properties were manipulated. In the *strong gun* condition, the gun fired with perfect accuracy, the bullets dealt substantial damage, and the rate of fire was moderate and steady. To augment the player's perception of the gun's strength, the gun's firing animation and sound were further modified: the gun fired with a subtle flash of light, the screen shook slightly and the sound was deep and powerful. Enemies shot by the powerful rifle would send gobbets of blood into the air and across the walls and floor, and when killed, would burst into gore, losing chunks of flesh or even limbs. The powerful rifle was capable of slaying most enemies in one or two shots. The degree of screen-shake and muzzle flash was modest, not affecting the player's ability to aim or see.

In the *weak-gun* condition, the gun fired with dramatically poorer accuracy, the bullets dealt substantially less damage, and the rate of fire was slightly slower. The gun did not shake the screen when fired, and its sound was anaemic. Enemies shot by the rifle always died with the same, less dramatic animation, and did not lose blood or limbs when shot.

A reviewer expressed concern that these diverse properties involved in the gun power manipulation technically confounds some variables (accuracy, damage, rate of fire, graphics, sound and enemy animations). As this was the first experiment on this topic, we felt it necessary to use the strongest manipulation possible to see if any effect could be detected. If so, later experiments could study the contribution of the particular design elements to the *gestalt* effect.

A full table of statistics for the weak and strong guns is provided in table 1. Game files for all four conditions are publicly available in the GitHub repository.

**Table 1.** Properties of the strong and weak virtual weapons.

	gun condition	
	strong	weak
rate of fire (rounds per minute)	155	150
damage (hit points)	50	14
firing spread (degrees)	0.25	6
report	loud	modest
screen-shake	yes	no
muzzle flash	yes	no
enemies dismembered	yes	no

### 2.4. Outcomes

This being an initial, exploratory study, we included many potential outcomes. Some of these were direct, such as questions about the desirability of the real-world AR-15. Others were subtler and more circumscribed, such as questions about the safety of firearms, the importance of the Second Amendment, and potential restrictions on gun magazines.

#### 2.4.1. First and Second Amendment rights advocacy

Participants rated nine items on a seven-point Likert scale (1-*Strongly disagree*, 7-*Strongly agree*). Four items asked about the importance of the Second Amendment and the utility of private firearm ownership (these were summed; Cronbach's *α* = 0.79). The other five items asked about the importance of freedom of speech in violent media (also summed; *α* = 0.66); these were intended as distractor items.

#### 2.4.2. AR-15 desirability

At the top of the scale, participants were instructed that the following questions reference the AR-15 semi-automatic rifle. A picture of the rifle accompanied the text. Five questions measured participants' evaluations of the real-world AR-15. These questions asked whether the AR-15 would be fun to own, useful to own, would make the respondent feel safer, would be accurate, and would be powerful; these were summed (*α* = 0.74). Another three questions measured buying intentions (scale derived from [14], *α* = 0.84). A last question asked ‘What is the MOST you would be willing to pay, in dollars, for the AR-15?’ Participants wholly uninterested in owning an AR-15 were instructed to write ‘blank’ for this item.

#### 2.4.3. In-game gun desirability

Participants rated two items for how desirable their in-game gun was: ‘I feel I *want* the gun that I used in the video game today’ and ‘I feel I *need* the gun I used in the video game today.’ These were positively correlated (*r* = 0.41 [0.27, 0.52]), and so were summed for simplicity of presentation.

#### 2.4.4. Public policy

Five items measured attitudes towards gun control laws and the permissibility of carrying firearms in public; these were summed (*α* = 0.83).

#### 2.4.5. Normative gun safety and utility

Participants were asked what percentage of gun owners would ever experience a gun-related accident (e.g. accidental discharge), what percentage would ever have a gun stolen from them, and what percentage would ever use their gun in an act of self-defence.

#### 2.4.6. Magazine restrictions

Participants were asked what should be the maximum number of bullets in a magazine, that is, how many bullets a gun should be able to fire before needing to be reloaded.

#### 2.4.7. Individual differences

Participants were asked whether they owned a gun, whether they played violent video games, and which political party they supported.

## 3. Results

### 3.1. Analysis

The primary hypothesis motivating this study was that playing a video game featuring a realistic AR-15 would influence gun attitudes relative to playing the same game using a science-fiction weapon. Players who used a powerful and attractive AR-15 were expected to have more positive attitudes towards the real-world AR-15 and possibly stronger pro-gun opinions for public policy, relative to the science-fiction-weapon control. Players who used a weak and unattractive AR-15 were expected to have more negative attitudes towards the real-world AR-15 relative to the science-fiction-weapon control. We therefore tested the evidence for a 2 (gun type) × 2 (gun power) interaction.

We used the BayesFactor package [15] for R [16] to perform all analyses involving those outcomes that were normally distributed. This being some of the first research in the area, we did not know exactly what effect size to anticipate; thus, we used a default two-tailed Cauchy prior with scale *r* = 0.5, reflecting anticipated effects of modest size, commensurate with most effects in social psychology. For each outcome, we conducted an ANOVA with factors of gun type, gun power, participant's gender and participant's political orientation.

For each Bayesian analysis, we report Bayes factors. Bayes factors represent the ratio of the probability of the data given one model over the probability of the data given another model. This gives the evidence, in odds, for one model over another model. Multiplying this Bayes factor against the prior odds gives an updated posterior odds. For example, if one thought there was only a 1 in 10 chance that Manipulation A would have an effect, and the Bayes factor (BF) for a Manipulation A model over the null model was 10 : 1, the posterior odds of Manipulation A having an effect would be 1 : 1. That is, given an unlikely phenomenon and a considerable amount of evidence, the phenomenon would be considered as likely as not.

For each analysis, we compare four models. The first is a covariates-only model, which describes outcomes as a function of subjects' gender and political orientation. This model treats the experimental manipulations of gun type, gun power and their interaction as having had no effect. The second is a full model consisting of the covariates, the main effects of gun type and gun power, and the interaction of gun type and gun power. The third model is an additive model similar to the full model, but it removes the interaction of gun type and gun power. The fourth model is the null model, in which no variable predicts the outcome. Comparisons of the second model (full model) against the third model (additive model) give the evidence for or against our hypothesized effect, the gun type × gun power interaction.

Our percentage-based outcomes were better described with a gamma distribution. As BayesFactor does not allow comparison of generalized linear models, we simply report parameter estimates and *p*-values for these outcomes. Similarly, magazine capacity was not normally distributed and could not be analysed using BayesFactor. Readers with greater modelling acumen than ours are invited to retrieve the data from the GitHub repository and perform the appropriate Bayesian analysis.

#### 3.1.1. Manipulation check

We tested how assignment to the 2 × 2 design influenced participants' in-game performance, as measured by the number of times the player died and the number of monsters the player killed.

Count of player deaths was highly skewed and perhaps best described by a zero-inflated negative binomial distribution. A logit link was used for the zero inflation, and a log link was used for the negative binomial. Supporting this modelling decision, the overdispersion parameter was statistically significant, *p* = 0.003. With regard to the number of times players died, neither the gun power (strong versus weak; *b* = −0.031, *t*_170_ = −0.132, *p* = 0.895) nor gun type (realistic versus sci-fi; *b* = −0.091, *t*_170_ = −0.466, *p* = 0.642) significantly influenced this outcome. Gun power and gun type did not significantly interact (*b* = 0.043, *t*_170_ = 0.131, *p* = 0.896). With regard to whether players died at all (e.g. the zero-inflation model parameters), neither the gun power (*b* = 11.624, *t*_170_ = 0.065, *p* = 0.948) nor gun type (*b* = −2.538, *t*_170_ = −0.004, *p* = 0.997) had a significant effect. They did not significantly interact, either (*b* = 2.45, *t*_170_ = 0.004, *p* = 0.997).

Count of killed enemies was also highly skewed, and so a negative binomial distribution was fitted using a log link. Participants in the powerful-gun condition killed substantially more enemies than did those in the weak-gun condition (*b* = 0.798, *t*_170_ = 10.527, *p* < 0.001), supporting the efficacy of the gun-power manipulation. Neither gun type (*b* = −0.048, *t*_170_ = −0.632, *p* = 0.527) nor the gun type × gun power interaction (*b* = −0.045, *t*_170_ = −0.419, *p* = 0.675) were significantly related to the number of enemies killed.

Regrettably, we did not ask participants directly about how fun, powerful, satisfying, etc. the in-game gun was. As such, we do not have direct evidence that the powerful gun was more pleasant to use than the weak gun. At best, we might infer that, because the powerful gun was more effective, it was also more pleasant to use.

**Figure 2.**
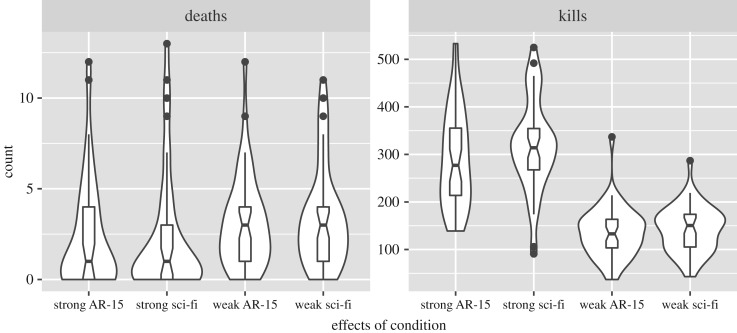
Distribution of manipulation checks. Participants in the powerful-gun condition died fewer times and killed more monsters. Notch width represents standard error of median.

**Figure 3.**
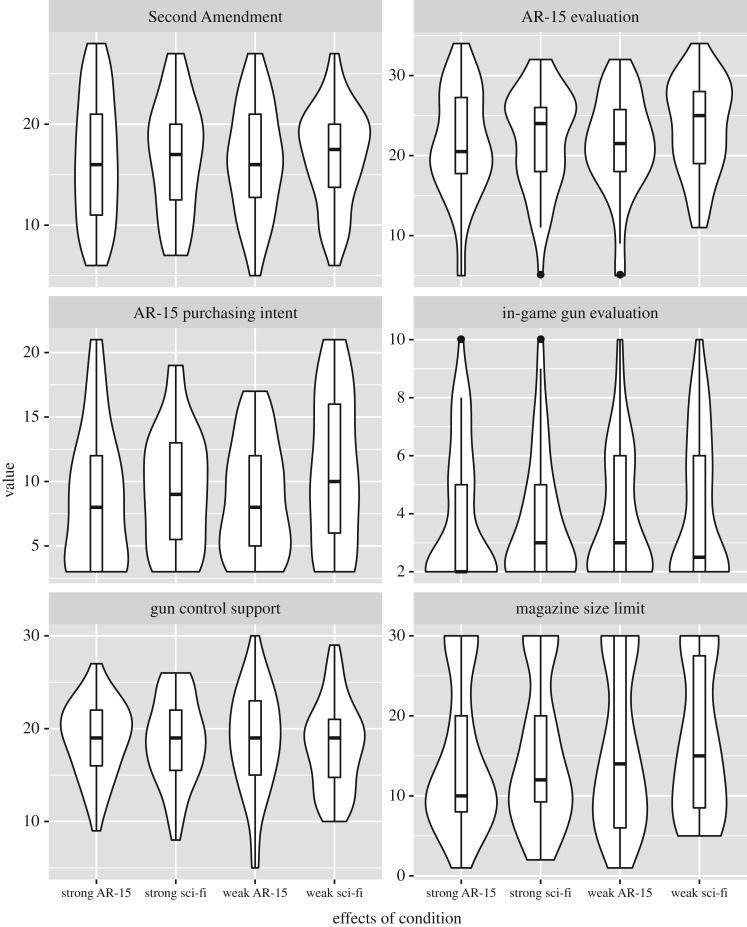
Distribution of product evaluation and gun control opinion outcomes. Distributions appear largely invariant across conditions.

**Figure 4.**
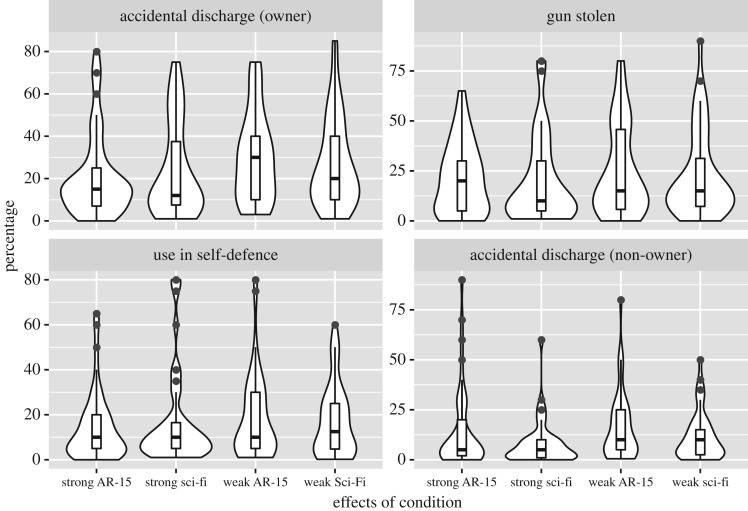
Perceived percentage rates of certain gun-related incidents across conditions. Responses are highly variable; covariates offered little in the way of prediction.

Combination violin/boxplots for each outcome are summarized in figures 2–4.

#### 3.1.2. Second Amendment advocacy

Participants' Second Amendment advocacy was best modelled by a simple additive model of political orientation and gender, BF = 7.33 × 10^4^ : 1 over the null. The covariates-only model was preferred over the full model (that is, the model with covariates, gun type, gun power and the gun type × gun power interaction), BF = 130 : 1. This indicates that the experimental condition had little explanatory power over and above that of the covariates. Comparison of the full model against the additive model let us examine the evidence for or against the hypothesized gun type × gun power interaction. The evidence was against this interaction, BF = 1 : 4.03 for the full model relative to the additive-effects model.

#### 3.1.3. Product attitudes

Again, attitudes towards the AR-15 were best described by a simple additive model of political orientation and gender, BF = 3.26 × 10^5^ : 1 over the null. The covariates-only model was preferred to the full model, BF = 49.8 : 1. The evidence was against a gun type × gun power interaction, BF = 1 : 3.18 for the full model relative to the additive-effects model.

#### 3.1.4. Purchasing intentions

Purchasing intentions were right-skewed. However, the QQplot of standardized residuals was not terribly misshapen, and transforming intentions by square root or logarithm did not improve the QQplot much. Thus, we analysed this outcome in its natural units with the standard general linear modelling technique.

Purchasing intentions were best described by additive effects of political orientation and gender, BF = 3.46 × 10^6^ : 1 over the null. The covariates-only model was preferred to the full model, BF = 19.6 : 1. The evidence was against a gun type × gun power interaction, BF = 1 : 2.14 for the full model relative to the additive-effects model.

#### 3.1.5. Desire of in-game weapon

This variable was very badly right-skewed, with most participants choosing the minimum response. Square-root or log transformation did little to fix this. We report this in its natural units, but readers with better ideas for modelling are encouraged to use the raw data to perform further tests.

Weapon desire was best described by an effect of gender, BF = 6.8 : 1 over the null. Adding the main and interactive effects of gun type and gun power to this gender-only model was not preferred, BF = 1 : 145. The evidence was against the predicted gun type × gun power interaction, BF = 1 : 4.4 for the full model relative to the additive-effects model.

#### 3.1.6. Policy opinion

Policy views were best described by political orientation and gender, BF = 8.75 × 10^9^ : 1 over the null. Adding the main and interactive effects of gun type and gun power was not supported, BF = 1 : 93.2. The evidence was against the hypothesized gun type × gun power interaction, BF = 1 : 4.35 for the full model relative to the additive-effects model.

#### 3.1.7. Rates of gun accidents and gun use

Participants' estimated rates seemed to be more appropriately modelled as a gamma distribution than a normal distribution. Because responses of 0% cannot be modelled under this distribution, these responses were adjusted to 0.001%. In general, we note that participants' estimates were highly variable, ranging from 0% to 80% or more.

Only a few idiosyncratic predictors reached statistical significance. Republicans, relative to liberals, thought it more probable that a gun owner would experience a gun-related accident such as an accidental discharge (*b* = 0.017, *t*_166_ = 2.668, *p* = 0.008). Men, relative to women, thought it more probable that a gun owner might have a gun stolen from them (*b* = 0.015, *t*_166_ = 2.373, *p* = 0.019). Libertarians, relative to other political parties, thought it more probable that a gun owner would ever use their gun in an act of self-defence (*b* = 0.138, *t*_166_ = 2.28, *p* = 0.024). None of these estimated rates were significantly predicted by the game participants had played. Full tables of model output are provided in electronic supplementary material, table S1.

#### 3.1.8. Magazine capacity

Several participants listed very large values (e.g. 100 or more) for a maximum magazine size, or wrote in responses to the effect that there should be no such government-imposed limit. We tried modelling this outcome in two ways. First, we winsorized all responses in excess of 30 down to 30 and attempted a linear model. Second, we coded a dichotomous variable for responses less than 30 and responses equal to or greater than 30 and attempted a logistic model. The linear model suggested that males (*b* = 3.508, *t*_163_ = 2.229, *p* = 0.027) and libertarians (*b* = 5.579, *t*_163_ = 2.032, *p* = 0.044) supported larger magazine sizes than did females and democrats. The logistic model found no significant predictors. Neither model detected any significant effects of game.

## 4. Discussion

Results indicate that brief use of a realistic (versus science-fiction) firearm in the context of a fantasy violent video game does little to influence attitudes towards that firearm or to firearms more generally. In all of the models and for all of the outcomes we considered, the gun type × gun power interaction explained very little variance. The observed magnitude of this coefficient was more consistent with the null hypothesis than with a reasonable alternative hypothesis. Participants' political orientation, and often their gender, strongly accounted for their views of firearms. The best models estimated the effects of these demographic factors while treating the experimentally manipulated game features as irrelevant.

In general, men, conservatives and libertarians, when compared with women and liberals, had more positive feelings towards guns, expressed greater subjective value for guns, and indicated more positive attitudes towards the AR-15. The experiment's manipulation of video game content did not affect these outcomes. These results indicate that attitudes towards guns may be better predicted by relatively stable personal traits than by transient influences of brief video game exposure.

### 4.1. Limitations and future directions

There are a number of possible reasons we did not detect an effect. The simplest explanation, of course, is that no such effect exists: product placement in violent games might have only a minimal influence on attitudes towards those products. However, this would seem a little surprising given the broader phenomenon as reported in news outlets and summarized in our introduction.

One major limitation is that the study did not involve a no-violence control condition. Perhaps the players' participation in any form of gun violence is enough to shape attitudes towards the broader outcomes such as Second Amendment advocacy, policy views, and perceptions of gun safety. Even so, one would expect the specific portrayal of a powerful AR-15, relative to a less-powerful AR-15 or a science-fiction weapon, might shape opinions towards the AR-15 specifically. That we failed to observe such an effect suggests that these effects, if they exist, are either smaller than expected, apply only to some subset of the population with a particular constellation of traits, or require a different methodology.

Some issues may have limited our ability to detect an effect. First, it is possible that 15 min is not enough to influence attitudes towards a gun. Second, it is possible that the game's setting was not conducive to product-placement effects, being too fantastic for the real-world weapon. Perhaps a more realistic setting such as an urban neighbourhood or American countryside would influence attitudes more so than did our game's unrealistic hellish landscape populated by zombies and demons. Such a setting might be particularly effective given a heroic scenario, such as stopping a home invasion or mass shooter; this would provide a strong test of the hypothesis. Third, perhaps participants already had relatively crystallized attitudes towards the AR-15. Given the AR-15's prominent role in the Newton mass shooting that had preceded our experiment by only a few months, perhaps participants had generally decided before the experiment whether they favoured or disfavoured the AR-15. Fourth, perhaps limiting the player to only one gun reduced the effect of the manipulation; differences in the target gun's strength might be more salient when a second control gun is present to be compared against.

Additionally, some research suggests that violent content may distract from in-game ads [17]. As knowledge of the particular gun brand was not necessary for effective game performance, participants may have allocated attention away from the gun brand. By contrast, games such as *Call of Duty* invite players to select a loadout of particular weapons, each having different properties, strengths and weaknesses that have substantial influences on gameplay. In this sort of game environment, greater attention might be paid to the gun's name and brand.

Future work could address these weaknesses through the following endeavours. First, experimental manipulations could involve longer gameplay sessions for a more powerful manipulation. Second, researchers might use and modify games with more realistic settings and enemies. Perhaps instead of *Doom*, which takes place in a science-fiction hellscape, researchers might use and modify a *Grand Theft Auto* game to have the desired weapon with the desired properties, as *Grand Theft Auto* takes place in a more realistic setting. Third, the game might do more to encourage attention to the name of the gun, perhaps by including several firearms and manipulating the strength of a branded exemplar. Fourth, it could be enlightening to study the effects of video game gun violence in general, rather than the effects of a specific embedded product, on attitudes towards guns. Lastly, researchers may wish to study firearm-product-placement effects in non-experimental paradigms such as cross-sectional or longitudinal surveys. A comprehensive and scientific survey study could help to confirm or refute the product-placement phenomena described by media.

All the same, we conclude that product-placement effects of violent video games on the desire to own guns may be smaller, subtler and harder to detect than we had personally anticipated. This conclusion is strengthened by our use of the strongest manipulation we could design, combining practical properties (damage, accuracy and rate of fire) and presentational properties (sound, graphics and animation) to make the gun seem powerful or weak.

## 5. Conclusion

Attitudes towards firearms are likely to be determined largely by political views. Political views are often strong and resistant to change. Relative to the influence of political beliefs, the effects of brief exposure to a violent game featuring an attractive firearm may be minimal. If product placement in video games does change attitudes towards firearms in general or towards a brand of firearm specifically, these changes may be smaller and subtler than we anticipated, may require more sophisticated experimental procedures to study, may require longer exposure periods for cultivation or may be limited to some subset of the general population.

## Supplementary Material

Model output regarding rates of gun accidents and gun use.

## Data accessibility

Our data and code are deposited at GitHub: https://github.com/Joe-Hilgard/VVG-product-placement.
